# Crystal structure reveals vaccine elicited bactericidal human antibody targeting a conserved epitope on meningococcal fHbp

**DOI:** 10.1038/s41467-018-02827-7

**Published:** 2018-02-06

**Authors:** Jacinto López-Sagaseta, Peter T. Beernink, Federica Bianchi, Laura Santini, Elisabetta Frigimelica, Alexander H. Lucas, Mariagrazia Pizza, Matthew J. Bottomley

**Affiliations:** 1 GSK Vaccines srl, Via Fiorentina 1, 53100 Siena, Italy; 2 Immunobiology and Vaccine Development, UCSF Benioff Children’s Hospital, 5700 Martin Luther King Jr. Way, Oakland, CA 94609 USA; 3 GSK Vaccines, 14200 Shady Grove Road, Rockville, MD 20817 USA

## Abstract

Data obtained recently in the United Kingdom following a nationwide infant immunization program against serogroup B *Neisseria meningitidis* (MenB) reported >80% 4CMenB vaccine-mediated protection. Factor H-binding protein (fHbp) is a meningococcal virulence factor and a component of two new MenB vaccines. Here, we investigated the structural bases underlying the fHbp-dependent protective antibody response in humans, which might inform future antigen design efforts. We present the co-crystal structure of a human antibody Fab targeting fHbp. The vaccine-elicited Fab 1A12 is cross-reactive and targets an epitope highly conserved across the repertoire of three naturally occurring fHbp variants. The free Fab structure highlights conformational rearrangements occurring upon antigen binding. Importantly, 1A12 is bactericidal against MenB strains expressing fHbp from all three variants. Our results reveal important immunological features potentially contributing to the broad protection conferred by fHbp vaccination. Our studies fuel the rationale of presenting conserved protein epitopes when developing broadly protective vaccines.

## Abstract (web summary)

Factor H binding protein (fHbp) is a meningococcal virulence factor and a component of vaccines against serogroup B *Neisseria meningitidis*. Here, the authors characterize the vaccine-elicited human antibody Fab 1A12 and present both the free and the fHbp-bound Fab 1A12 crystal structures.

## Introduction

Meningococci cause fatal cases of bacterial sepsis and meningitis, with serogroup B (MenB) strains particularly prevalent in Europe^1,2^. Two vaccines based on protein antigens were developed for the prevention of MenB disease. One of these antigens is factor H-binding protein (fHbp), which was identified independently by reverse vaccinology using genomic sequences^3^ and by traditional methods using biochemical fractionation^4^. FHbp elicits protective antibody responses in mice, rabbits, rhesus macaques^3,5,6^, and humans^7–9^. The vaccines are referred to as 4CMenB (Bexsero; GSK) and Bivalent rLP2086 (Trumenba; Pfizer) and both are licensed for use in adolescents in the United States. Only 4CMenB is licensed for infants starting 2 months of age in Europe, Canada, Australia, and several countries in South America. Of note, following a nationwide implementation of 4CMenB, a recent study showed >80% vaccine-mediated protection against all current MenB strains in the United Kingdom^10,11^.

Antibodies to fHbp elicit protection through complement-mediated bactericidal activity^3,4^. Some antibodies also inhibit the binding of human complement factor H (fH) to the bacteria, rendering them more susceptible to complement^12^. While some antibodies to fHbp elicited in mice inhibited the binding of fH to the bacterial surface^12,13^, the antibodies elicited in rhesus macaques^14,15^ or humans^16^ generally did not inhibit binding of fH. This difference may result from the inability of murine fH to bind fHbp^16^, in contrast to human fH that binds fHbp, such that the dynamics of epitope exposure, dependent on fH binding, are likely different when immunizing mice and humans.

Bactericidal polyclonal antibodies raised in mice were reported to be mainly directed against the carboxyl (C)-terminal domain of fHbp^17^. Epitope mapping of murine anti-fHbp monoclonal antibodies (mAbs) has confirmed that many of the amino-acid residues involved in antibody binding are located in the C-terminal domain^17–19^. There are several examples, however, of epitopes involving residues in the amino (N)-terminal domain^20–23^. Detailed epitope-mapping studies of anti-fHbp mAbs have been performed using nuclear magnetic resonance spectroscopy^18,22^, hydrogen-deuterium exchange followed by mass spectrometry^21,24^, and by X-ray crystallography^24,25^. The latter studies recently defined a mechanism by which two murine anti-fHbp antibodies (mAbs JAR5 and 12C1) may synergize to elicit complement-mediated bactericidal activity^25,26^. Moreover, both mAbs target epitopes that overlap with the fH-binding site^24,25^, thus revealing the structural basis for their inhibition of fH binding. Structural epitope-mapping studies with murine Fabs have also been performed for another protective antigen present in 4CMenB, namely the outer membrane protein PorA^27–29^.

In an important recent study, the human antibody repertoire to fHbp was investigated for the first time, by characterization of a panel of 10 human anti-fHbp antibody fragments (Fabs) cloned from three subjects vaccinated with 4CMenB^16^. Therein, two of the three subjects raised broadly reactive antibodies (termed 9B and 10C). Fab 9B (hereafter termed Fab 1A12) was of particular interest since it bound with extremely high affinity (*K*_D_ = 19 pM) to fHbp variant 1.1 (var1.1) and, moreover, cross-reacted with all eight fHbp sequence variants tested, including representatives from all three phylogenetic variant groups. This Fab was particularly unusual because most known antibodies against fHbp are “variant group-specific”, i.e., most mAbs efficiently bind fHbp from one variant group, but not from both the other two variant groups. Indeed, despite previous analyses of hundreds of mAbs raised against fHbp by animal immunizations, only a few have been reported to exhibit some cross-reactivity, including MN86-994-11^30^, JAR41^23^, 17C1^21^, and 30G4^21^. Within the fHbp variant groups, amino-acid sequence identity is usually above 87%; whereas, between variant groups the sequence identity can fall to as little as 62%, and this high antigenic variability presumably underlies the rarity of eliciting cross-reactive mAbs^3,23,30^.

The observations summarized above raise the question: “What is the structural basis of the broad antigen-recognition properties of the vaccine-elicited human antibody 1A12?” Since meningococci display enormous antigenic diversity (~ 1000 sequence variants of fHbp have been reported^31^), it is important to understand how current MenB vaccine antigens interact with the human immune system. Such details are expected to provide insights into vaccine efficacy and may enable the design of next-generation vaccines.

In this study, we present the crystal structures of the broadly reactive Fab 1A12 alone and in a complex with fHbp, thereby elucidating the structural basis for the antigen-recognition properties of this human antibody. We also show that Fab 1A12 as an intact IgG antibody has high affinity for different fHbp variants, and for point mutants, revealing the contribution of specific amino acids in the epitope recognized by the human antibody. Finally, in functional assays, IgG 1A12 has bactericidal activity. These data provide the crystallographic and functional characterization of a functional vaccine-elicited human antibody targeting a bacterial pathogen.

## Results

### Human mAb 1A12 shows affinity and broad reactivity for fHbp

**Fig. 1:**
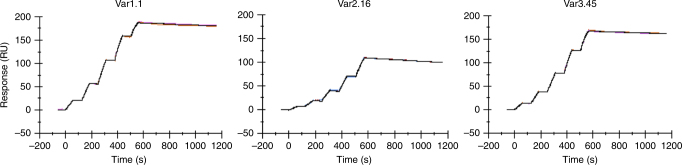
mAb 1A12 shows high-affinity cross-reactive binding to fHbp in SPR studies. In each panel, sensorgrams show the experimental association and dissociation traces (colored) performed in duplicate for the binding of the different fHbp subvariants to captured mAb 1A12; the calculated fitting traces are shown in dark gray. Full kinetic analyses of each interaction are reported in Table 1

Fab 1A12 derives from an adult human subject immunized with a MenB vaccine formulation that contained fHbp var1.1 (see Methods). The cross-reactivity of recombinant Fab 1A12 in enzyme-linked immunosorbent assay (ELISA) experiments using the three different variant groups of fHbp was reported previously^16^. To extend those investigations, here we used mammalian cells to produce 1A12 as an intact full-length mAb of the IgG1 subclass (the subclass most abundant in human sera), and *Escherichia coli* to produce recombinant fHbp antigens. Surface plasmon resonance (SPR) was used to determine the kinetics for immobilized mAb 1A12 binding to solution phase fHbp antigens representative of the three different variant groups: fHbp var1.1; fHbp var2.16; and fHbp var3.45. All three variants were recognized by mAb 1A12, as indicated by the sub-nanomolar equilibrium dissociation constant (*K*_D_) values of 87, 384, and 138 pM for fHbp var1.1, var2.16, and var3.45, respectively (Fig. 1 and Table 1).

**Table 1:** Binding kinetic values determined for mAb 1A12 by surface plasmon resonance

	Var1.1	Var2.16	Var3.45	Var1.1 A162P	Var1.1 G163A	Var1.1 G163N	Var1.1 K180A	Var1.1 K185A	Var1.1 N190A	Var1.1 N215G
*k*_on_ (M^−1^ s^−1^) × 10^5^	6.2 ± 0.1	2.3 ± 0.01	4.2 ± 0.01	10.1 ± 0.8	6.3 ± 0.02	8.3 ± 1.0	3.3 ± 0.01	1.5 ± 0.02	4.7 ± 0.2	8.1 ± 0.04
*k*_off_ (s^−1^) × 10^−5^	5.4 ± 0.7	8.7 ± 0.5	5.7 ± 0.3	2.4. ± 0.9	2.7 ± 0.2	4.6 ± 0.7	0.9 ± 0.2	32.1 ± 1.8	175.8 ± 7.9	50.2 ± 0.7
*K*_D_^a^ (pM)	87 ± 10	384 ± 24	138 ± 7	24 ± 11	44 ± 3	55 ± 2	28 ± 6	2158 ± 149	3713 ± 11	620 ± 12

^a^*K*_D_ = *k*_off_/*k*_on_; mean and SD values were calculated from SPR experiments performed in duplicate for each fHbp variant and mutant

### Structure determination of human Fab 1A12 bound to fHbp

**Fig. 2:**
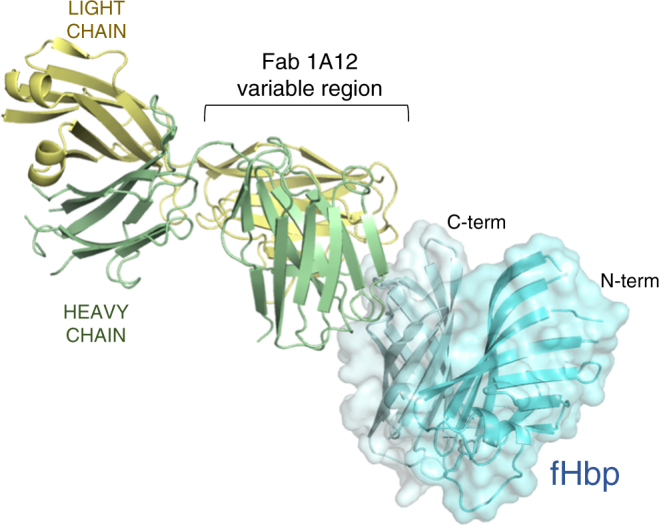
The Fab 1A12-fHbp complex crystal structure. Ribbon diagram in which the heavy and light chains of Fab 1A12 are colored green and yellow, respectively; fHbp is represented in cyan with a transparent surface. Artwork was prepared using PyMOL

Since mAb 1A12 was raised by vaccination with fHbp var1.1, we sought structural information to explain its cross-reactivity and the precise recognition mode of its epitope. We obtained crystals of Fab 1A12 bound to fHbp var1.1 that initially diffracted to 3.5 Å resolution. By an iterative streak-seeding approach, we subsequently obtained better diffracting crystals (belonging to space group P2_1_) and ultimately determined the structure via molecular replacement with a resolution of 2.2 Å (*I*/*σI* = 0.98, CC½ = 0.26 in the highest resolution shell^32^, see Methods and Table 2). Two Fab/fHbp complexes were present in the asymmetric unit and were essentially identical, exhibiting a root mean square deviation (rmsd) of 0.5 Å across all alpha carbon atoms. The overall structure of the complex shows Fab 1A12 projecting all six complementarity-determining region (CDR) loops onto a surface-exposed region at one end of the C-terminal β barrel of fHbp, while the N-terminal region of fHbp does not contribute to the interaction (Fig. 2). Overall, 17 fHbp residues are involved in a curved interface. The buried surface area on fHbp is 800 Å^2^, which is typical for Fab/antigen complexes^33,34^. Fab 1A12 binds fHbp with a major contribution from the heavy chain, and a minor contribution from the light chain (590 Å^2^ vs. 210 Å^2^). The binding interface comprises charged, polar, and van der Waals (VDW) interactions.

**Table 2:** X-ray data collection, processing, and refinement statistics

	Fab 1A12-fHbp complex	Fab 1A12 alone
Resolution range (Å)	48.91–2.20 (2.27–2.20)	70.88–1.76 (1.82–1.76)
Space group	P 1 2_1_ 1	P 3_1_ 2 1
Unit cell dimensions		
*a*, *b*, *c* (Å)	42.82 163.95 110.66	131.90 131.90 90.38
*α*, *β*, *γ* (°)	90.0 97.7 90.0	90.0 90.0 120
Total reflections	414 763 (25 038)	1 615 701 (132 068)
Unique reflections	74 237 (5623)	88 113 (8430)
Multiplicity	5.6 (4.5)	18.3 (15.6)
Completeness (%)	96.0 (73.0)	97.0 (93.0)
Mean *I*/sigma(*I*)	6.98 (0.98)	33.18 (1.68)
Wilson *B*-factor	27.4	22.3
Rmerge	0.194 (1.193)	0.155 (2.534)
Rmeas	0.214 (1.353)	0.170 (2.827)
CC1/2	0.987 (0.263)	0.919 (0.185)
R-work	0.192 (0.307)	0.199 (0.347)
R-free	0.250 (0.355)	0.223 (0.355)
Number of atoms		
Macromolecules	9848	3497
Ligands	13	0
Protein residues	1318	444
RMS bonds (Å)	0.003	0.007
RMS angles (°)	0.58	0.91
Ramachandran favored (%)	97	96.8
Ramachandran allowed (%)	3.2	3.2
Ramachandran outliers (%)	0.077	0.0
Average *B*-factor	22.23	27.62
Macromolecules	22.01	27.03
Ligands	21.47	n/a
Solvent	24.55	34.30

Values in parentheses are for highest resolution shell. ${\rm Rmerge} = \frac{{\mathop {\sum}\nolimits_{hkl} {\mathop {\sum}\nolimits_{i = 1}^n {\left| {I_i\left( {hkl} \right) - \bar I\left( {hkl} \right)} \right|} } }}{{\mathop {\sum}\nolimits_{hkl} {\mathop {\sum}\nolimits_{i = 1}^n {I_i\left( {hkl} \right)} } }};{\rm Rmeas} = \frac{{\mathop {\sum}\nolimits_{hkl} {\sqrt {\frac{n}{{n - 1}}} } \mathop {\sum}\nolimits_{i = 1}^n {\left| {I_i\left( {hkl} \right) - \bar I\left( {hkl} \right)} \right|} }}{{\mathop{\sum}\nolimits_{hkl} {\mathop {\sum}\nolimits_{i = 1}^n {I_i\left( {hkl} \right)} }}}$

**Fig. 3:**
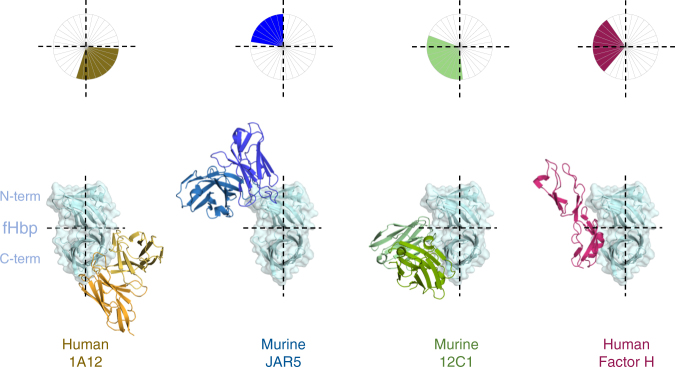
Fab 1A12 shows a unique binding mode. Bottom: surface and ribbon representations of fHbp, bound to 1A12 (yellow), JAR5 (blue), 12C1 (green), and factor H (red). For clarity, only the Fab variable regions are shown. Top, schematic diagram of the different binding sites on fHbp

The Fab 1A12-binding site on fHbp is completely different from the two structurally characterized epitopes of the murine Fabs 12C1 and JAR5^24,25^, which are both specific only for fHbp variant group 1 antigens. To compare the modes of binding to fHbp, we conceptually divided the fHbp molecule into quadrants by drawing “crosshairs” on its long and short axes, thus creating a reference frame (Fig. 3). While both JAR5 and 12C1 target the left half of fHbp, and in particular the upper (N-terminal) and lower (C-terminal) quadrants, respectively, 1A12 binds fHbp on its lower right quadrant, in a distinctly new region (Fig. 3). Similarly, the 1A12-binding site does not overlap that of human factor H, which binds on the two left quadrants of fHbp^35^, thus providing the molecular explanation for previous observations that Fab 1A12 does not inhibit binding of fHbp to factor H^16^.

### Details of a cross-reactive conformational epitope on fHbp

**Fig. 4:**
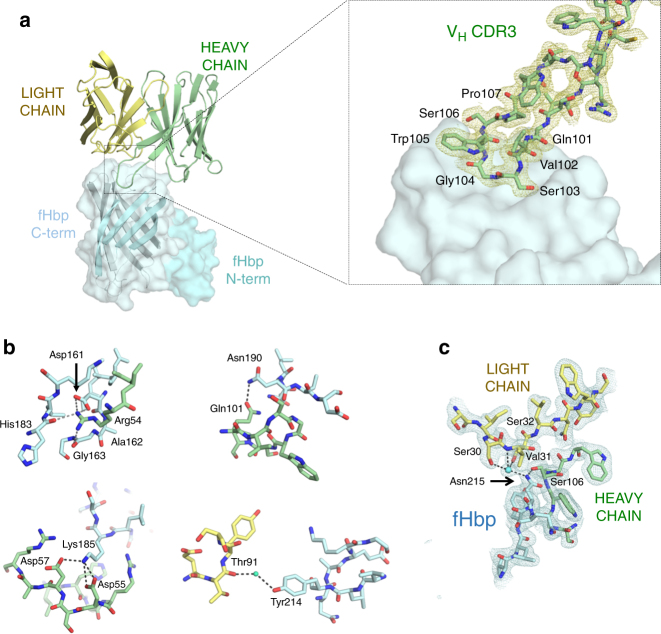
Intermolecular interactions in the Fab 1A12/fHbp-binding interface. **a** Left: ribbon representation highlighting the region where the Fab V_H_ CDR3 loop contacts fHbp. The N- and C-terminal domains of fHbp are displayed in surface mode in different blue palette colors; the Fab is colored as in Fig. 2. For clarity, the constant regions of the Fab have been omitted. Right: the V_H_ CDR3 loop (stick bonds) and its 2Fo-Fc electron density map (yellow mesh) at 1*σ* contour level. Fab constant regions are omitted for clarity. **b** Noteworthy salt bridges and other polar interactions at the binding interface, involving V_H_ CDR2 and 3. (FHbp: cyan; Fab light chain: yellow; Fab heavy chain: green). **c** The binding interface centered around fHbp residue Asn215 is shown as sticks. Polar interactions (≤3.3 Å) established with the heavy and light chains are represented by dashed lines. The cyan sphere represents a water molecule. The blue mesh depicts the 2Fo-Fc electron density map associated with the region displayed, plotted at 1*σ* contour level

A close inspection of the Fab 1A12/fHbp-binding interface reveals a predominant role in antigen recognition for the Fab heavy chain, and especially for the heavy chain variable (V_H_) CDR3 loop which extends into a notable groove on the fHbp surface (Fig. 4a). In the V_H_ CDR3 loop, all residues from Q101 to P107 (except V102) act to secure an extensive network of backbone and side-chain polar and VDW contacts, and presumably all contribute to the extremely tight interaction with the antigen (Fig. 4a and Supplementary Table 1). In addition, several other striking contacts are established via salt bridges between Asp161 on fHbp and Arg54 on the heavy chain (Fig. 4b, upper left), and Lys185 on fHbp and Asp55/Asp57 on the heavy chain (Fig. 4b, lower left), and, via hydrogen bonds between Asn190 on fHbp and Gln101 on V_H_ CDR3 (Fig. 4b, upper right). Further, a water-mediated hydrogen bond is formed between Thr91 in the light chain CDR3 and Tyr214 on fHbp (Fig. 4b, lower right). Importantly, Asn215 on fHbp simultaneously contacts both the heavy and light chains of Fab 1A12, by hydrogen bonding with the gamma oxygen atoms of three serine residues (heavy chain Ser106 directly, and light chain Ser30 and Ser32 indirectly through water-mediated interactions) and with Val31 (backbone nitrogen) on the light chain (Fig. 4c).

**Fig. 5:**
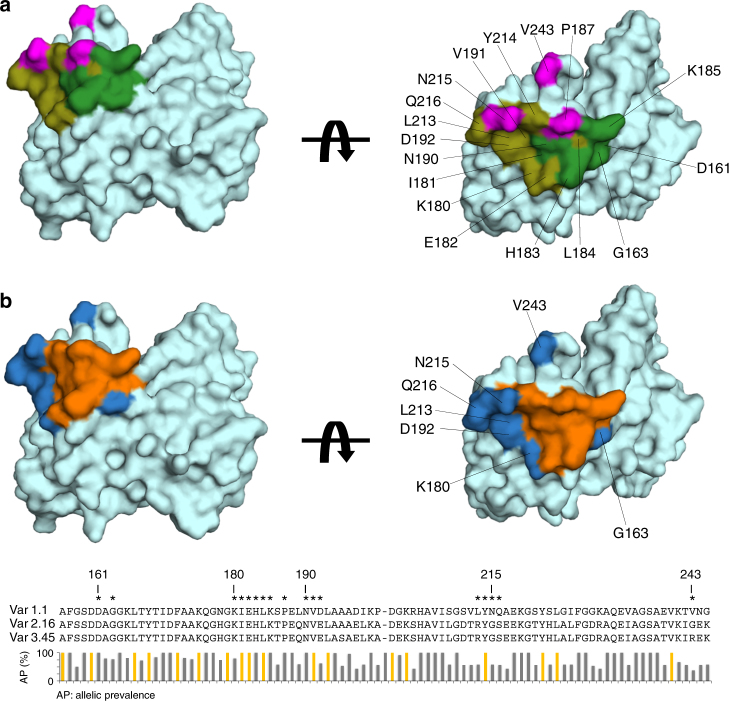
The 1A12 epitope and its allelic diversity in the fHbp global gene repertoire. **a** Two views of the 1A12 epitope “footprint” on the surface of fHbp. Residues contacted by the heavy chain are highlighted in green and olive colors for polar and VDW interactions, respectively. The contacts made by the light chain are in magenta. Asn215 establishes polar contacts with both the heavy and light chains. **b** Allelic diversity in the 1A12 epitope. Upper panel: residues within the 1A12 epitope with a degree of conservation >99% in all fHbp gene repertoire are colored orange; residues with a prevalence lower than 99% are shown in dark blue and labeled with their position number. Bottom panel: sequence alignment of fHbp var1.1, 2.16, and 3.45. (The gap at position 200–201 reflects one subvariant of 984 that presents a single-residue insertion (Trp) at this position. Despite the gap, the numbering shown above the alignment corresponds to the numbering used in the main text). The allelic prevalence among 984 fHbp sequences is shown for each position in the 1A12 epitope^31^. Orange columns depict sites non-polymorphic in all 984 sequences known. The residues that form the 1A12 epitope are indicated with an asterisk

A surface representation of all the fHbp residues that interact with 1A12 reveals the nature of the conformational epitope on fHbp, lying on a surface-exposed well-ordered region of the C-terminal β barrel. The epitope is concentrated in a cluster of residues targeted by the V_H_ CDR2 and CDR3 loops, and a more isolated area contacted by the light chain (Fig. 5a).

### Basis of 1A12 cross-reactivity despite antigenic diversity

The elucidation of the present structure allows us to provide a detailed molecular explanation for the versatility of mAb 1A12 to recognize fHbp antigens from all three variant groups. Remarkably, many of the fHbp residues that participate in the interaction with the Fab (12 of the 17 residues in the 1A12 epitope) are conserved across the three different fHbp variants tested here by SPR, i.e., var1.1, var2.16, and var3.45 (Fig. 5b). Notably, residues Asp161 and Asn190 are completely conserved in fHbp variants 1.1, 2.16, and 3.45, and play key roles in the overall network of interactions with the Fab (Fig. 4b). Further, the motif _180_KIEHLK_185_, and residues Asn190, Val191, and Tyr214 are also conserved in the same three variants tested by SPR. Therefore, the degree of conservation assigns a leading role to these residues in the cross-recognition by the human mAb 1A12.

The *Neisseria* Multi Locus Sequence Typing database now contains ~ 1000 different polypeptide sequences for fHbp obtained from naturally occurring strains^31^. Therefore, we performed a deeper analysis in silico and calculated the degree of conservation associated with residues in the 1A12 epitope in 984 fHbp sequence variants available to date, which include sequences from serogroup B strains and from other serogroups^31^. Most notably, five residues (Ile181, Glu182, Leu184, Val191, and Tyr214) are 100% conserved throughout the whole fHbp sequence repertoire (Fig. 5b). Furthermore, five additional epitope residues show ≥99% conservation (Asp161, His183, Lys185, Pro187, and Asn190). Together, these observations suggest that mAb 1A12 might display cross-reactivity with a vast breadth of recognition across almost the entire known polymorphic repertoire of fHbp.

### Effects of polymorphisms in the 1A12-fHbp-binding interface

**Fig. 6:**
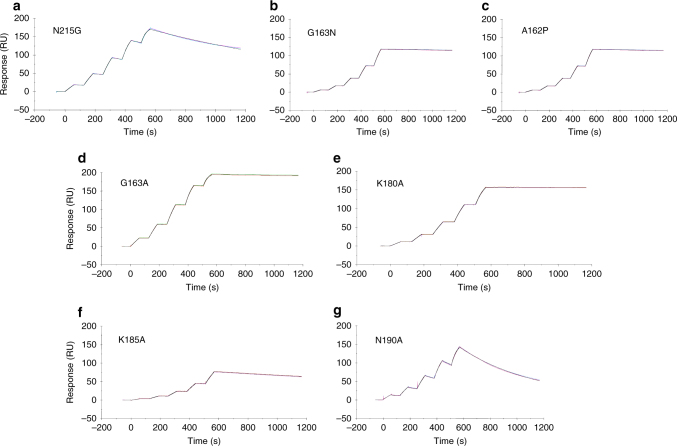
Impact of fHbp var1.1 site-specific mutations on the binding to mAb 1A12. The effect of the mutations was analyzed by SPR with mAb 1A12 captured on the surface, and the association and the dissociation of diverse mutants (panels **a**-**g**) were monitored in duplicate (colored traces). The single-cycle kinetics fitting (Langmuir 1:1 model) is represented as solid black lines in each sensorgram (full details of kinetic analyses are provided in Methods and Table 1)

To better define the contribution of individual residues and the effect of polymorphisms within the epitope, we made single-amino-acid substitutions in the fHbp var1.1 background: A162P; G163A; G163N; K180A; K185A; N190A; and N215G. The fHbp Asn215 residue makes contacts directly or indirectly with six different residues in the heavy and light chains of Fab 1A12 (Fig. 4c and Supplementary Table 1). Substitution of Asn215 with Gly (N215G), as found in var2.16 and var3.45, resulted in a notable decrease in binding to mAb 1A12 (Fig. 6a). However, given the extremely tight binding of mAb 1A12 to wild-type var1.1, the seven-fold decrease in affinity due to the N215G mutation nevertheless resulted in a very tight antigen/antibody interaction (*K*_D_ = 620 pM, compared to 87 pM for wild type).

The fHbp residue Gly163 is also found in the epitope/paratope interface, using its backbone carbonyl oxygen to contact the important heavy chain residue Arg54 (Fig. 4b). It was conceivable that this backbone-mediated interaction coupled with the small size of Gly163 (and of its neighbor Ala162) were key factors in the optimal accommodation of the Arg54 side chain from 1A12. However, the single mutations A162P and G163N (both polymorphisms naturally occurring at this position in some variant 2 and 3 proteins) actually resulted in mild increases in binding affinity toward mAb 1A12 (Fig. 6b, c). fHbp var1.1 G163A and K180A mutants also slightly increased the binding affinity (approximately two- to threefold), thus suggesting that the side-chain characteristics in these positions are not crucial determinants for cross-recognition by mAb 1A12 (Table 1 and Fig. 6d, e**)**. These results contribute to a better understanding of the molecular basis of this interaction and the potential overall cross-reactivity of mAb 1A12, since G163 and K180 show lower prevalence throughout all strains (G163 is found in ~ 50% of subvariants; K180 in ~ 70% of subvariants), despite both being conserved in var1.1, var2.16, and var3.45. In summary, known polymorphisms occurring in fHbp at positions 163 and 180 are unlikely to preclude binding of mAb 1A12 to meningococcal strains expressing such antigens.

Finally, we studied the roles of Lys185 and Asn190. The former establishes salt bridges with two acidic residues on the Fab heavy chain, Asp55 and Asp57, and VDW contacts with His52 and Arg54 (Fig. 4b and Supplementary Table 1) while the latter is directly involved in hydrogen bonds with Gln101 and Gly104 and VDW contacts with Ser103. In SPR studies, mutant K185A reduced the binding affinity to 1A12 by ~ 25-fold (Fig. 6f and Table 1). Similarly, N190A notably impacted the binding to 1A12 with an ~ 50-fold reduction in the affinity (Fig. 6g and Table 1). While both the association and dissociation rates are affected in the case of K185A, it is predominantly the increased dissociation rate (*k*_off_) that is responsible for the lower affinity of N190A. These results confirm the importance of these highly conserved positions on fHbp recognition by 1A12.

In conclusion, the very high affinity of mAb 1A12 for wild-type fHbp var1.1 appears to be the result of a cooperative and elaborate network of interactions. Despite the sequence diversity inherent to fHbp, we show here that mAb 1A12 recognizes a series of fHbp variants with very high affinities, suggesting a high breadth of coverage potentially conferred by this human mAb.

### Structure of free Fab 1A12 reveals paratope flexibility

**Fig. 7:**
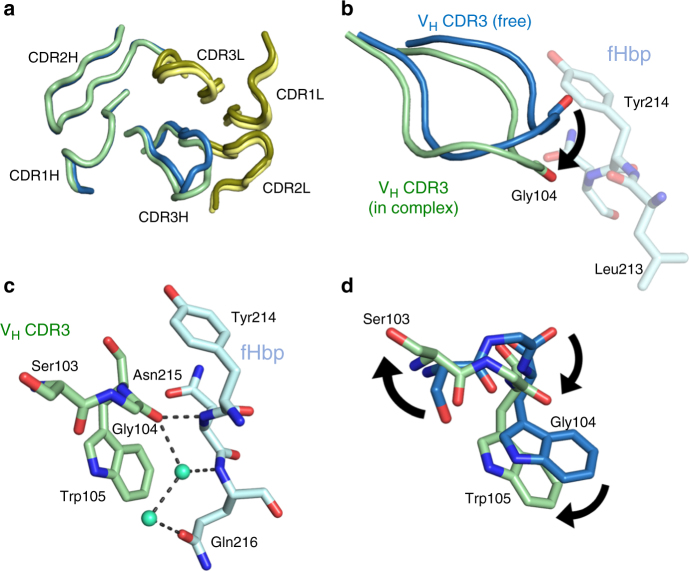
Conformational changes between bound and free Fab 1A12. **a** Ribbon diagram showing the light (dark and light yellow) and heavy chains (green and blue) of Fab 1A12 both in the liganded (pale colors) and unliganded (dark colors) states. Only CDR3H shows a notable difference. **b** V_H_ CDR3 loop conformations are represented as cartoons with colors distributed in a similar manner to **a**; fHbp residue is colored cyan. The movement of Gly104 is indicated. **c** Detail of the Gly104 region in the bound state. **d** Side chains of Ser103 and Trp105 show notably different positions in bound and free forms

We also determined the crystal structure of the free Fab 1A12 at 1.76 Å resolution (Table 2). Comparison of the free and antigen-bound Fab structures shows that they are highly similar (rmsd 0.69 Å on alpha carbons). However, superposition reveals that while most of the CDR loops do not change their conformation (Fig. 7a), there is a difference in the V_H_ CDR3 loop conformation upon complex formation. Most notably, Gly104 in V_H_ CDR3 shifts position by 4 Å, thus avoiding a steric clash with Tyr214 on fHbp (Fig. 7b).

In the complex, Gly104 establishes polar and water-mediated contacts with fHbp residues Asn215 and Gln216 (Fig. 7c). Similarly, the neighboring V_H_ CDR3 residues Ser103 and Trp105 also show changes of varying magnitude in their side-chain positions (Fig. 7d), enabling them to make favorable contacts with fHbp. On the other side of the interface, when compared with free fHbp^36^, it emerges that upon binding most fHbp residues do not change conformation. One exception is a short loop (fHbp residues 241–246), wherein the alpha carbon of Val243 moves by 3 Å and its side chain undergoes a rotation of ~ 90° thereby optimizing contacts with Fab 1A12.

### mAb 1A12 recognizes diverse fHbp variants on MenB surface

**Fig. 8:**
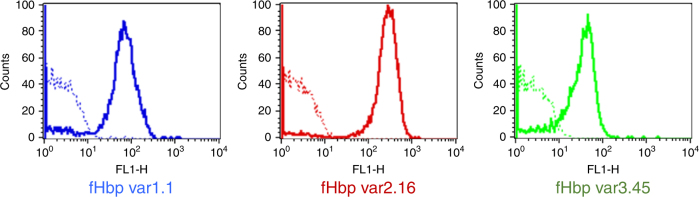
mAb 1A12 binds meningococci expressing all three fHbp variant groups. Flow cytometry histograms showing the binding of mAb 1A12 to live serogroup B meningococci H44/76, M08-0240104, and M01-0240320 strains (blue, red and green lines, respectively) when incubated with 10 μg ml^−1^ of anti-fHbp mAb. Dotted-line histograms represent negative control, bacteria incubated with PBS and anti-human IgG FITC-conjugated

We sought to understand how the broad cross-reactivity of 1A12 relates to the function of this antibody. We used 1A12 as an intact human IgG1 mAb and examined its binding to live bacteria by flow cytometry. We observed that mAb 1A12 binds to all three tested MenB strains expressing fHbp from different variant groups: strains H44/76 (fHbp var1.1); M08-0240104 (fHbp var2.16); and M01-0240320 (fHbp var3.45). The var2.16-expressing strain showed the strongest binding, whereas slightly lower levels of binding were observed with the var1.1- and var3.45-expressing MenB strains (Fig. 8). The order of binding affinities found by SPR and the degree of binding observed via flow cytometry analysis were different. Assuming that technical differences (between SPR and flow cytometry) do not underlie these observations, we interpret the discrepancy as suggesting that factors other than affinity may affect the overall extent of mAb binding to the live bacterial cells; for example, the antigen density displayed on the bacterial surface. Indeed, the M08-0240104 strain was previously reported to have high expression of fHbp var2.16, whereas the var1.1 and var3.45 strains were reported to express approximately two- to fourfold lower amounts of fHbp antigen (Supplementary Table 2)^37^. Nevertheless, these findings confirm the results of SPR analyses in a physiologically more relevant context (live bacterial cells), showing that there is broad cross-recognition by mAb 1A12 despite extensive fHbp sequence variability and likely numerous other phenotypic differences existing between diverse meningococcal strains.

### Bactericidal activity of cross-reactive mAb 1A12

Finally, we investigated whether mAb 1A12 was bactericidal against MenB strains expressing different fHbp variants, in an assay measuring complement-dependent killing (using baby rabbit serum as the complement source). We selected this assay to assess the functional activity of the mAb because the serum bactericidal assay (SBA) is the widely accepted functional correlate of protection for *Neisseria meningitidis*^38–40^. Meningococcal strains H44/76 (expressing fHbp var1.1), M08-0240104 (expressing fHbp var2.16), and M01-0240320 (expressing fHbp var3.45) were incubated with different concentrations of mAb 1A12. Indeed, mAb 1A12 induced bactericidal activity against all three different strains. The concentrations of mAb 1A12 required for ≥50% killing of bacteria when measured against strains expressing fHbp var3.45, var2.16, and var1.1 were 0.06, 0.49, and 3.9 μg ml^−1^, respectively. Consequently, 1A12 is not only immunologically cross-reactive, but most importantly, it is cross-protective, being able to elicit rabbit complement-dependent bactericidal activity against meningococci in all three of the fHbp variant groups tested herein.

## Discussion

Vaccines against infectious diseases save an estimated 2–3 million lives each year^41^. Research now targets the development of new vaccines against diseases not yet preventable by current vaccines, and also aims to enhance the breadth of coverage of some current vaccines against highly mutable pathogens. Upon vaccination, protein antigens are recognized by host immune factors (surface-bound B-cell receptors, or soluble antibodies) and elicit protection usually via an antibody-mediated regulatory, or killing, immune response. Therefore, high-resolution structural data of antibody–antigen complexes facilitate an understanding of the molecular bases of immunoprotection, and may consequently aid the development of optimal vaccine immunogens^42–44^. Here we sought structural insights into the human immune recognition of fHbp, a key antigen in the two licensed MenB vaccines.

We determined the crystal structure of fHbp var1.1 bound to the Fab fragment of a vaccine-elicited human mAb, 1A12. Despite having been generated in a human subject immunized with the fHbp sequence variant 1.1, the mAb 1A12 binds with remarkably high affinity (sub-nanomolar *K*_D_) to representatives from each of the three distinct variant groups of fHbp, and is therefore an antibody of great interest. Our structural and biochemical insights reveal the molecular basis of this cross-reactivity. Previous reports of cross-reactive anti-fHbp mAbs were limited to those raised in mice, and mapped their epitopes to N-terminal regions^21,23^, or did not provide any epitope-mapping information^30^. An important implication of our findings is that human immunization with fHbp var1.1 in the 4CMenB vaccine may contribute to confer an unexpectedly broad coverage against meningococci expressing fHbp from any of the three known variant groups.

To our knowledge, this is the first report of a vaccine-elicited human Fab bound to a bacterial antigen. One recent report described crystal structures of two human Fabs obtained from memory B cells of healthy donors, and described an unusual mode of recognition of a staphylococcal antigen predominantly mediated by V_H_ CDR2^45^. Here the structure of the 1A12/fHbp var1.1 complex shows how the hypervariable V_H_ CDR3 loop interacts with a groove containing several discontinuous residues clustered on a highly solvent-exposed region of the fHbp C-terminal β barrel domain. Overall, the recognition of the antigen by Fab 1A12 is governed by polar interactions. Numerous H-bonds, salt bridges, water-dependent contacts, and VDW interactions are widely distributed across the binding interface and contribute collectively to the very strong recognition of fHbp. This cross-reactive conformational epitope presents a unique binding mode that was not previously seen in other crystal structures of fHbp complexed with mAbs raised in mice^24,25^, nor in additional murine mAbs reported to target epitopes on the N-terminal domain of fHbp^21,23^. Further, comparison of the 1A12 epitope and the fH-binding site on fHbp^35^ reveals two quite distinct interaction areas, and thus provides the structural basis for the lack of inhibition of factor H binding to fHbp by human mAb 1A12, and also confirms that fHbp does not undergo notable conformational changes upon binding to either partner.

Recognition of fHbp by 1A12 does not follow the classical “lock and key” concept of antigen–antibody interactions. Rather, although fHbp var1.1 appears relatively rigid, the flexible V_H_ CDR3 loop of Fab 1A12 undergoes a notable conformational change, which allows the formation of several favorable interactions with fHbp. The V_H_ CDR3 sequence composition features small residues (Gly and Ser) and a large aromatic residue (Trp), which in itself is not unusual for a V_H_ CDR3^46^. The availability of both free and antigen-bound states of a Fab^47,48^ allows inspection at high resolution of the functionality in the paratope. In 1A12, the presence of Gly and Ser may promote flexibility and allow the versatile aromatic residues to mediate numerous interactions with epitope atoms, thus enabling antigen recognition^49^. In short, it appears that the distinct sequence composition of Fab 1A12 enables a structural transition in V_H_ CDR3, which translates into an energetically favorable antigen-binding region ideally suited to bind fHbp.

A detailed analysis of the antibody/antigen interface reveals how mAb 1A12 can be vastly cross-reactive. In short, of the total 17 fHbp epitope residues that make contact with the Fab, 12 are absolutely conserved, and a further 4 are conserved moderately (≥66%), in fHbp var1.1, var2.16, and var3.45. The high conservation of key epitope residues explains the ability of mAb 1A12 to cross-react with the different fHbp variants (either as purified fHbp proteins or when expressed on the surface of live meningococci). Moreover, even when a key fHbp epitope residue was mutated to remove its side-chain functionality (N215G), tight binding to mAb 1A12 was still observed (sub-nanomolar *K*_D_ value). Furthermore, other naturally occurring fHbp substitutions (A162P and G163N) actually increased the strength of mAb binding. These observations suggest that the epitope/paratope interface defined by 1A12 can also accommodate at least some known sequence polymorphisms without losing binding functionality.

A vast number of fHbp sequence variants identified from clinical isolates and carrier strains are now known^31^. Therefore, we also analyzed the conservation of the 1A12 epitope residues in the 984 subvariants reported to date. We found that several epitope residues are absolutely conserved (5 of 17 residues) throughout the entire fHbp antigenic repertoire, and an additional 5 residues have extremely high (≥99%) prevalence. Therefore, 10 of 17 epitope residues are at least 99% conserved in the known antigenic repertoire. Although additional investigations would be needed to demonstrate the full cross-reactivity of mAb 1A12 toward the many known subvariants, we envisage a wide recognition of the great majority of fHbp antigens, with potential to induce bacterial killing either alone or cooperatively with other mAbs against fHbp or in synergy with antibodies against alternative MenB surface antigens. The observation that antibodies recognizing ordered conformational epitopes are less sensitive to antigen sequence diversity than those antibodies targeting disordered epitopes^33^ further underscores the likelihood that mAb 1A12 may react with most fHbp variants.

We found that mAb 1A12 bound tightly to all three variants of fHbp when tested in biochemical assays (SPR), and live cell-based binding assays (flow cytometry). Interestingly however, we found that the binding affinities determined with the soluble recombinant proteins did not correlate closely with the amount of binding to whole bacteria as measured by flow cytometry (Table 2). Using the selected reaction monitoring mass spectrometry approach, the surface concentration of fHbp in these strains was previously determined to be ~ 4000, 9000, and 1000 molecules per cell for variants var1.1, var2.16, and var3.45, respectively^37^. Given that mAb 1A12 showed very high affinity for each fHbp subvariant, it is likely that the higher antigen density on var2.16 underlies the higher fluorescence response measured here for the var2.16 strain.

Importantly, mAb 1A12 not only recognized the three fHbp variants on the surface of live meningococci, it was also able to activate the complement cascade and induce bacterial killing against MenB strains expressing fHbp var1, var2, or var3 antigens, as demonstrated in bactericidal assays, here using baby rabbit serum as the complement source. That is, mAb 1A12 is cross-protective as well as being cross-reactive. Some anti-meningococcal mAbs have bactericidal activity only when combined with other mAbs targeting different epitopes or even different antigens on the same bacterial cell. In contrast, mAb 1A12 is able to induce the killing alone (with higher efficiencies for var2 and var3 strains), presumably through highly efficient activation of the classical pathway of the complement system, which highlights the benefit of immunologically targeting this epitope on fHbp. Somewhat counter-intuitively, we observed that the killing activity was strongest against the M01-0240320 (fHbp var3.45) strain, where the surface density of fHbp is the lowest. It is possible either that the M01-0240320 strain is inherently more susceptible to killing, or that the particular fHbp var3.45 antigen density on this strain was sterically or geometrically more efficient for mAb 1A12-dependent activation of the complement pathway, or both. While the susceptibility to complement-mediated killing (using polyclonal anti-fHbp sera in the SBA assay) has been shown to correlate with the absolute amount of fHbp protein expressed by each meningococcal strain^37^, some additional strain-specific differences in the intrinsic susceptibility to killing by unique mAbs are likely determined by other factors, such as the expression of virulence molecules that bind host complement regulators^50^.

The most efficient complement-dependent immune response against a specific surface antigen may result from the activity of two or more different mAbs engaging the same antigen simultaneously^25^. In general, it is not the action of only one mAb but the combination of different mAbs in a polyclonal response that are directed against alternative non-competing epitopes that will act cooperatively to maximize the efficiency of the immune response^51^. Therefore, the cross-protective human mAb 1A12 characterized here appears to be a potentially key player in such a multivalent bactericidal response. The extent to which such a cross-reactive mAb could contribute to meningococcal killing in vivo in a vaccinated individual may depend on its IgG subclass and will of course also depend on the absolute quantity in which the mAb is present^52^. While it was beyond the scope of this study to determine the serum concentrations of individual mAbs, recently published proteomic approaches combined with next-generation sequencing have demonstrated that a molecular deconvolution of the immune response can be performed^53^, and this might form the basis of future studies to further explore the response to meningococcal vaccines such as 4CMenB.

In summary, we present here the crystal structures of an fHbp-specific human Fab in free and antigen-bound states, elicited by vaccination. We define a molecular signature that allows a vaccine-elicited human mAb to cross-react with the three different variants of fHbp and importantly, to induce complement-dependent killing responses against MenB strains harboring fHbp antigens from variants 1, 2, or 3. The existence of this cross-protective epitope on fHbp var1.1 suggests that the broad efficacy demonstrated by the 4CMenB vaccination in the United Kingdom^10,11^ could result from a multi-factorial effect, where antigens carrying cross-protective epitopes play key synergistic roles. Moreover, such detailed structural studies could be exploited for the design of vaccines with an immunofocusing approach toward cross-protective epitopes, aiming to further enhance the existing breadth of protection. More broadly, it is noteworthy that several current vaccines against bacterial pathogens are essentially based on surface-exposed polysaccharides that make up the outermost layer of the bacterial surface. However, when capsular polysaccharides are unsuitable vaccine candidates, or when polysaccharide serotypes are too numerous and variable, alternative reverse and structural vaccinology approaches may permit the identification and design of protein-based epitope-focused vaccine candidates. In this light, our studies provide an exploratory human vaccination model enabling the identification of broadly protective epitopes that could be expanded to the design of ideal saccharide-independent cross-protective bacterial targets.

## Methods

### Human samples

Human peripheral blood mononuclear cells were collected from three vaccinated subjects 8 days after the second dose of a multicomponent serogroup B meningococcal vaccine containing recombinant fHbp variant 1.1. Plasmablasts were processed individually (not pooled), were isolated as single cells, and were used as the source from which genes of heavy and light chain variable regions were amplified separately and then combined by overlap extension PCR, in order to obtain Fab region sequences. Recombinant Fabs were then produced in *E. coli* (see Protein expression description below) and were screened for antigen specificity through ELISA assays involving detection of fHbp-bound Fabs by Fab-specific goat anti-human IgG conjugated to alkaline phosphatase (Sigma; 1:5000 in phosphate-buffered saline (PBS)-Tween 20-bovine serum albumin (BSA)^16^. The samples were obtained from a clinical trial conducted in Krakow, Poland, in a study sponsored by Novartis Vaccines & Diagnostics, now part of the GSK group of companies. The clinical trial protocol was approved by the Bioethics Committee of the District Medical Doctors’ Chamber in Krakow and the study was conducted in accordance with the Declaration of Helsinki. Written informed consent was obtained from each of the subjects.

### Protein expression

All genes for the preparation of the fHbp subvariants and point mutants used in this study were cloned and amplified using DH5α and MultiShot™ StripWell Mach1™ T1 Phage-Resistant Chemically Competent *E. coli* cells (Invitrogen), expressed from pET vectors (Novagen) induced by Isopropyl-β-d-thiogalactoside in *E. coli* strain BL21 (DE3) (New England Biolabs) and purified via C-terminal 6-His tags using Ni^2+^-affinity chromatography columns (His-Trap HP, 1 ml, GE Healthcare) and buffer solutions recommended by the manufacturer, controlled by an ÄKTA Purifier liquid chromatography system (GE Healthcare). PCR primers used to generate fHbp point mutants are listed (Supplementary Table 3). Full-length factor H was purchased from Calbiochem. For the expression of mAb 1A12, the variable regions of the heavy and light chains of 1A12 were codon-optimized (Supplementary Table 4) for expression in mammalian cells and synthesized by GeneArt (Thermo Fisher). Synthetic DNA sequences were digested with *Eco*RI (New England Biolabs) and cloned into the human pRS5a expression vectors encoding the Igγ1 and Igκ backbone, under the control of the cytomegalovirus promoter and in frame with a leader sequence for secretion derived from human immunoglobulins (Novartis-NIBR). The recombinant antibody was transiently expressed in Expi293 cells by transfecting the cells with equivalent amounts of both plasmids with the use of the Expi293 expression system (Thermo Fisher). Three and six days after transfection, cells were harvested, centrifuged for 10 min at 350 ×* g*, and filtered through a 0.2 μm filter to remove cellular debris. Recombinant antibody was purified from the tissue culture expression medium with Protein G Sepharose 4 Fast Flow (GE Healthcare), following the manufacturer’s protocol. A PD-10 Desalting Column (GE Healthcare) was used for buffer exchange and the antibody was eluted in PBS pH 7.4. 1A12 IgG concentration was determined in a NanoDrop spectrophotometer (Thermo Scientific) and its purity was assessed by SDS-PAGE on a 4–12% Bis-Tris Gel and Problue Safe Stain (Giotto Biotech).

The recombinant plasmid for human Fab 1A12 and the expression in *E. coli* (New England Biolabs) have been previously described^16^. The bacteria were suspended in 50 mM NaH_2_PO_4_, 500 mM NaCl, 20 mM imidazole, pH 7.0, and lysed using chicken egg white lysozyme, DNase, and RNase (Sigma; 0.1 mg ml^−1^ each), and three freeze/thaw cycles. The clarified lysate was applied to a HiTrap Chelating HP (5 ml; GE Healthcare) column and the bound protein was eluted with an imidazole gradient from 20 to 250 mM. The Fab was further purified by cation exchange chromatography (HiTrap SP HP 5 ml; GE Healthcare) using 20 mM sodium acetate buffer, pH 5.5, and elution with a NaCl gradient from 0.02 to 1.0 M. Fractions containing the Fab were dialyzed against 20 mM Tris-HCl, 20 mM NaCl, pH 7.0 for crystallization trials.

For formation of the complex, fHbp var1.1 was expressed and purified as described above. Fab-expressing *E. coli* cells were first sonicated in ice-cold 10 mM HEPES (pH 7.4) and 150 mM NaCl, and centrifuged at 9500 × *g* for 30 min. The supernatant was then filtered and loaded on a Ni^2+^ Sepharose 6 Fast Flow column (GE Healthcare) pre-saturated with recombinant fHbp var1.1. The bound protein was eluted with 10 mM HEPES (pH 7.4), 150 mM NaCl, and 300 mM imidazole. Next, the protein was subjected to three cycles of concentration and dilution with 10 mM HEPES (pH 7.4) and 150 mM NaCl using an Amicon concentrator (Millipore) with a 30 kDa cutoff. The complex was then recovered for crystallization assays.

### Surface plasmon resonance

All interaction experiments were performed using a BIAcore T200 instrument (GE Healthcare), equilibrated at 25 °C. First, the mAb 1A12 was captured to a density of ~ 540 resonance units on the surface of a CM5 sensor chip previously coated with covalently immobilized monoclonal mouse anti-human IgG (Fc) antibody (GE Healthcare). In order to subtract the background signal for kinetic analysis, we prepared a control reference channel in a similar way but in the absence of the mAb. A series of concentrations of the different fHbp variants (wild type or mutants) were then injected in 0.01 M HEPES (pH 7.4), 0.15 M NaCl and 3 mM EDTA. To minimize nonspecific interactions, the running buffer was also supplemented with 0.005% v/v surfactant P20 and 0.1 % (w/v) BSA. All kinetic parameters were calculated using standard single-cycle kinetics fitting and a Langmuir 1:1 binding model (BIAcore T200 evaluation software).

### Crystallization and diffraction data

The purified Fab (7.1 mg ml^−1^) was screened against 96 crystallization reagents (Index Screen, Hampton Research). Crystals were observed in multiple conditions; after 4 days, the largest crystals were found in 60% Tacsimate, pH 7.0. Optimization of the crystallization conditions was achieved in 57.5% Tacsimate (Hampton Research) at pH 6.0. Crystals were soaked in 15% PEG 3350 and 12% Tacsimate as cryoprotectant and cryo-cooled in liquid nitrogen. The crystals were tested for diffraction at beamline 5.0.1 at the Advanced Light Source, Lawrence Berkeley National Laboratory and several data sets were collected on an ADSC Q315R detector (Table 2). Data were integrated and scaled with iMosflm^54^. The structure of the Fab was solved using Phaser^55^ with a homology model constructed from the 1A12 sequence and the coordinates of the human anti HIV 21c Fab (PDB ID 3LMJ) using Swiss-Model^56^.

The Fab 1A12-fHbp complex at 10 mg ml^−1^ was screened with a matrix of ~ 800 crystallization conditions using a Crystal Gryphon robot (Art Robbins Instruments). Long plate-needle-looking crystals were found at days 7–10 with 17% PEG 3350 and 0.2 M sodium malonate (pH 4.0). Optimization of the original conditions was performed by changing the concentrations of the different components in the initial crystallization mixture. Changes in the pH did not yield any improvement. Crystals were soaked in the original mother liquor supplemented with 10 % ethylene glycol as cryoprotectant and prior to cryo-cooling in liquid nitrogen. Diffraction of the crystals was tested at beamline ID29 of the European Synchrotron Radiation Facility and several full data sets were collected on a Pilatus 6M detector. Diffraction data sets were indexed, integrated, and scaled with XDS^57^ and Aimless^58^, via the CCP4 suite^59^. The structure of the complex was solved by molecular replacement with Phaser^55^ using as model templates for fHbp, light chain, and heavy chains the coordinates deposited in the protein data bank under the codes 2YPV, 4YPG, and 4JQI, respectively. The CDR3 loop of the heavy chain was omitted in the template.

### Structure refinement

Initial molecular replacement solutions were subjected to subsequent cycles of manual building in Coot^60^ and refinement with Phenix.refine^61^. The buried surface areas and atomic interactions/contacts, and the root mean square displacements, were calculated with PISA^62^ and Superpose^63^, respectively. All the structural figures were created with PyMOL^64^.

### Flow cytometry analysis

The ability of mAb 1A12 to bind the antigen exposed on the surface of *N. meningitidis* bacteria, expressing different fHbp variants, was determined using a FACScan flow cytometer. Bacteria grown until early log phase (OD600 of ~ 0.25) were incubated with mAb at the concentration of 10 µg ml^−1^. Antibody binding was detected using a goat anti-Human IgG conjugated to fluorescein isothiocyanate (Jackson Immuno Research, catalog number 109-096-088) at a 1:100 dilution. Bacteria plus PBS-1% BSA and secondary antibody were used as negative control.

### Bactericidal assay

Bacteria grown in Mueller Hinton broth supplemented with 0.25% glucose until early log phase (OD600 of ~ 0.25) were diluted in Dulbecco’s PBS containing 1% BSA and 0.1% glucose at the working dilution of 10^4^–10^5^ colony forming units (CFU) per ml and incubated with serial twofold dilutions of test mAb starting from a concentration of 31.25 µg ml^−1^ (corresponding to 1/16 dilution in the reaction mixture in the well). Bactericidal titers were defined as the reciprocal mAb dilution resulting in 50% decrease in CFU per ml after a 60-min incubation of bacteria with the reaction mixture compared to the control CFU per ml at time zero. Pooled baby rabbit serum (Cedarlane) was used as a complement source.

### Data availability

Structure factors and atomic coordinates have been deposited in the Protein Data Bank for the Fab 1A12 (ID 5UR8) and Fab 1A12-fHbp var1.1 complex (ID 5O14). Other data are available from the corresponding authors upon reasonable request.

## Electronic supplementary material

Supplementary Information
