# Supplementary material for: Crystal structure reveals vaccine elicited bactericidal human antibody targeting a conserved epitope on meningococcal fHbp
Source: Nat Commun. 2018 Feb 6;9:528. doi: 10.1038/s41467-018-02827-7 (PMC5802752; doi:10.1038/s41467-018-02827-7)
Supplement: Supplementary file 1 — Supplementary Information [file 41467_2018_2827_MOESM1_ESM.pdf]

Supplementary information for:

Crystal structure reveals vaccine elicited bactericidal human antibody targeting a conserved epitope on meningococcal fHbp

López-Sagaseta *et al*.

Supplementary Table 1. Atomic contacts between Fab 1A12 and fHbp var1.1

fHbp	1A12 Heavy chain	Bond Type
Asp161 $O^{\delta 1}$	Arg54 $N^{\eta 1}$, Arg54 $N^{\eta 2}$	Salt bridge
Gly163 O	Arg54 $N^{\eta 2}$	Hydrogen bond
Lys180	Ser103	Van der Waals
Ile181 O	Ser103 O^{γ}	Hydrogen bond
Glu182	Ser103	Van der Waals
His183 O	Arg54 $N^{\eta 1}$, Arg54 $N^{\eta 2}$	Hydrogen bond
His183	Asn31, Tyr32	Van der Waals
Leu184	Arg54	Van der Waals
Lys185	His52	Van der Waals
Lys185	Arg54	Van der Waals
Lys185N^{ζ}	Asp55 $O^{\delta 1}$, Asp55 $O^{\delta 2}$, Asp57 $O^{\delta 2}$	Salt bridge
Lys185 O	Trp33 $N^{\varepsilon 1}$	Hydrogen bond
Pro187	Pro107	Van der Waals
Pro187 O	Gln101 $N^{\varepsilon 2}$	Hydrogen bond
Asn190 $N^{\delta 2}$	Gln101 $O^{\varepsilon 1}$	Hydrogen bond
Asn190	Ser103	Van der Waals
Asn190 O	Gly104 N	Hydrogen bond
Val191	Gly104	Van der Waals
Asp192	Gly104	Van der Waals
Leu213	Gly104	Van der Waals
Tyr214	Gln101	Van der Waals
Tyr214	Gly104	Van der Waals
Tyr214	Trp105	Van der Waals
Tyr214	Ser106	Van der Waals
Tyr214	Pro107	Van der Waals
Asn215 N	Gly104 O	Hydrogen bond
Asn215	Trp105	Van der Waals
Asn215 $O^{\delta 1}$	Ser106 N	Hydrogen bond
Asn215 $N^{\delta 2}$	Ser106 O^{γ}	Hydrogen bond

Gln216	Trp105	Van der Waals

**fHbp**	**1A12 Light chain**	**Bond Type**
Pro187	Tyr97	Van der Waals
Asn215	Val31	Van der Waals
Asn215	Ala50	Van der Waals
Asn215 $N^{\delta 2}$	Ser32 O^{γ}	Hydrogen bond
Val243	Tyr92	Van der Waals

Supplementary Table 2. Comparison of affinities, cellular distribution and bactericidal activity across fHbp variants

	**mAb affinity (*K_D*, pM)**	**fHbp molecules/cell***	**Mean fluorescence intensity**	**Bactericidal mAb (μg.mL^{-1})**	**Bactericidal titer#**
**Var1.1**	87	4031	62	3.9	128
**Var2.16**	384	9390	238	0.49	1024
**Var3.45**	138	1111	31	0.06	8192

* Biagini, M. *et al.* Expression of factor H binding protein in meningococcal strains can vary at least 15-fold and is genetically determined. *Proc Natl Acad Sci USA* **113**, 2714-9 (2016).

# mAb dilution factor resulting in 50% decrease in colony forming units (CFU) per mL after a 60-min incubation of bacteria with the reaction mixture compared to the control CFU per mL at time zero. This column is an alternative way of showing the data contained in the preceding column, and simply shows the effective mAb dilution factor determined, in each case starting from a mAb stock at 0.5mg.mL^{-1} concentration.

Supplementary Table 3. PCR primer sequences for fHbp mutagenesis

**Primer name**	**Sequence**
741 v1.1 A162P For	gacgatcctggcggaaaactgacctacacc
741 v1.1 A162P Rev	tccgccaggatcgtctgaaccgaac
741 v1.1 G163A For	cgatgccgcgggaaaactgacctac
741 v1.1 G163A Rev	cagttttcccgcggcatcgtctgaaccg
741 v1.1 G163N For	gatgccaacggaaaactgacctacaccatag
741 v1.1 G163N Rev	ttttccgttggcatcgtctgaaccgaac
741 v1.1 K180A For	ggaaacggcgcgatcgaacatttgaaatcgc
741 v1.1 K180A Rev	gttcgatcgcgccgtttccctgcttggc
741 v1.1 K185A For	catttggcgtcgccagaactctcgccagaactc
741 v1.1 K185A Rev	ctggcgacgccaaatgttcgattttgc
741 v1.1 N190A For	cagaactcgcggtcgacctggccgcc
741 v1.1 N190A Rev	ggtcgaccgcgagttctggcgatttc
741 v1.1 N215G For	cctttacggccaagccgagaaaggcag
741 v1.1 N215G Rev	ggcttggccgtaaaggacggaaccgctgat

Supplementary Table 4. DNA sequences of 1A12 codon-optimized genes

1A12 Heavy Chain Sequence

gaggtgcagctggtgcagtctggcgccgaactgaagaaacctggggagagcctgaagatctcctgcaagg
ccagcggctacaccttcaccaactactgggtcgtgtgggtgcgccagatgcctggagaggggctcgaatgga
tgggctccatccacccccgggacagcgacgccagatacagcctgagcttcgagggcagagtgaccttcagc
gtggacaagagcaccaccaccgcctacctgcagtggtccagcctgaaagtcagcgacagcgccatctacta
ctgcgccagactgagccaggtgtccggctggtccccttgggtgggaccttggggccagggcaccctggtgac
cgtgtcctct

1A12 Light Chain Sequence

gacatcgtgatgacccagagccccagcagcctgagcgccagcgtgggagacagagtgaccatcacatgc
agagccagccagagcatcagcgtgtccctgaactggtatcagcagaagcctgggaaggcccctaaggtgct
gatctacgccgcctcccggctgcagagcggaatccccagcagattttctggcagcggcagcggctcccactt
caccctgaccatcagcagcctgcagcccgaggacttcgccacctactactgccaagaaacctacagcgacc
tgatgtacacattcggccagggcaccaaggtggaaatcaag

HEAVY CHAIN variable

EVQLVQSGAELKKPGESLKISCKASGYTFTNYWVVWVRQMPGEGLEWMGSIHPRDSDARYSLSFEGRVTFSVDKSTTT
AYLQWSSLKVSDSAIYYCARLSQVSGWSPWVGPWGQGTLVTVSS

CDR1: YTFTNYWVV
CDR2: WMGSIHPRDSDARY
CDR3: RLSQVSGWSPWVGP

LIGHT CHAIN variable

DIVMTQSPSSLSASVGDRVTITCRASQSISVSLNWYQQKPGKAPKVLIYAASRLQSGIPSRFSGSGSGSHFTLTISSL
QPEDFATYYCQETYSDLMYTFGQGTKVEIK

CDR1: QSISVSLN
CDR2: VLIYAASRLQS
CDR3: QETYSDLMY
